# Automatic detection and monitoring of abnormal skull shape in children with deformational plagiocephaly using deep learning

**DOI:** 10.1038/s41598-021-96821-7

**Published:** 2021-09-09

**Authors:** Seyed Amir Hossein Tabatabaei, Patrick Fischer, Sonja Wattendorf, Fatemeh Sabouripour, Hans-Peter Howaldt, Martina Wilbrand, Jan-Falco Wilbrand, Keywan Sohrabi

**Affiliations:** 1 Institute of Medical Informatics, Justus-Liebig University Giessen, 35392 Giessen, Germany; 2 Faculty of Health Sciences, University of Applied Sciences Giessen, 35392 Giessen, Germany; 3 Department for Cranio-Maxillofacial Surgery-Plastic Surgery, University Hospital Giessen, 35392 Giessen, Germany; 4 Department for Cranio-Maxillofacial Surgery-Plastic Surgery, Diakonie-Klinikum Jung-Stilling, 57074 Siegen, Germany

**Keywords:** Biomedical engineering, Paediatric research

## Abstract

Craniofacial anomaly including deformational plagiocephaly as a result of deformities in head and facial bones evolution is a serious health problem in newbies. The impact of such condition on the affected infants is profound from both medical and social viewpoint. Indeed, timely diagnosing through different medical examinations like anthropometric measurements of the skull or even Computer Tomography (CT) image modality followed by a periodical screening and monitoring plays a vital role in treatment phase. In this paper, a classification model for detecting and monitoring deformational plagiocephaly in affected infants is presented. The presented model is based on a deep learning network architecture. The given model achieves high accuracy of 99.01% with other classification parameters. The input to the model are the images captured by commonly used smartphone cameras which waives the requirement to sophisticated medical imaging modalities. The method is deployed into a mobile application which enables the parents/caregivers and non-clinical experts to monitor and report the treatment progress at home.

## Introduction

### Background

Nowadays, analysis of craniofacial anomalies representing a diverse group of deformities in the evolution of head and facial bones in newbies has become a multidisciplinary expertise domain^1^. Indeed, besides clinical experts and basic scientists, mental biologists and computer scientists have also been engaged in this domain. There are different factors like genetics, environment and, vitamin deficiency which might be involved in appearance and progress of the disease individually or in a combined form^2^. Craniofacial anomalies are categorized according to their ground reasons whether it is genetics or vitamin deficiency or environmental influence. There exist different types of craniofacial disorders in children; among them the most common types include cleft lip and palate, craniosynostosis and deformational plagiocephaly and brachycephaly. The other types as well as exact definition, subcategories and syndromes of each type can be studied by referring to the medical encyclopedias like^2^ since the medical context of the disease is not the main subject of this work. The prevalence of the disease in infected children varies depending on the craniofacial anomaly type. For example, 1 in 2000 of new births is affected by craniosynostosis and approximately 1 in 700 infants is born with cleft lip and/or cleft palate^3,4^. Abnormal head shape due to nonuniform expansion of skull results in several adversary effects in affected infants. Interactional hypertension, neurological complications like headache and developmental delay are some examples^3^. Therefore, early detection, treatment and quantifying the condition of craniofacial anomaly are of great importance not only due to patient-side concerns but also due to treatment success. Indeed, the early stage diagnosis is an essential factor in a timely-surgery to result in restoring the normal appearance of the child’s head shape by allowing cranial expansion^5^. Craniofacial disorders are normally diagnosed through clinical examination by trained craniofacial physicians. However, detection and classification of exact craniofacial type as well as quantifying the condition rely on more clinical methods like Computed Tomography (CT), plain radiography and morphometric evaluation^1,3,6–8^. The latter deals with head shape measurement in order to analyze and quantify the shape abnormality using anthropometric landmarks^9,10^. Although such a method and similar ones which require direct measurement of the child’s head are common clinical practices in treatment process, they all have a subjective nature which demands novel non-biased reliable methods in this regard. With the rapid progress in the field of computer vision, medical imaging modalities and artificial intelligence, and increasing computational power, the efforts towards designing and developing new less subjective methods have been escalated. Several methods have been proposed based on supervised machine learning and/or computer vision depending on the craniofacial anomaly types and corresponding sub-classes aiming at better understanding the disease condition or treatment progress. Depending on the craniofacial class type, these methods use 3D or 2D imaging modalities in order to automate the segmentation task in skull or facial bones assisting an accurate diagnosis or measurement required for sufficient treatment. The classical approaches based on machine learning require feature engineering performed on the preprocessed image data to feed the classifier(s). So, many methods have been proposed for the sake of feature generation and selection. For example, the proposed work in^11^ engaged the high dimensional distance matrix presentation of infant’s skull in order to classify different types of craniosynostosis. In another work, presented in^12^, 2D-histograms of surface normal vector angles have been extracted from 3D-mesh data for the sake of quantifying the severity and classifying deformational of deformational plagiocephaly. Other works like^5,13^ are based on some logistic regression techniques and lasso clustering classification for craniosynostosis classification on the extracted features from skull images. Recent advances in area of deep learning resulted in elaborate feature engineering carried out by the trained or pretrained networks. So, the extracted images feed the network aiming at classifying or segmenting the region of interest on 2D or 3D images.

### Presented work

In this paper, deformational plagiocephaly in children is the subject of our study. The methods based on computer vision and deep learning are presented for segmentation and quantification tasks in 2D image data captured by smartphone cameras in real-time. Our method achieves high accuracy in both tasks and facilitates the progress and treatment process of the patients to be cooperated by the parents or care givers at home. Preliminary results of this work have been presented in^14^ wherein the classification models and classification results have been introduced. In the current presented work, the model has been more elaborated and promoted, related literature has been studied, the data set has been augmented and extensive classification and regression analysis has been given. Finally, an app enabling capturing the photo of the babies head, classifying the head and measuring the corresponding parameters in real-time has been developed and presented.

### Related work

There is a large body of works in the literature addressing the classification problems in craniofacial diseases and corresponding syndromes based on computer vision, image processing and/or pattern recognition viewpoints. Here, the main aforementioned works concerning the analysis of deformational plagiocephaly leading to abnormal head shape in infants are focused. The statistical model of the head asymmetry in newborns with deformational plagiocephaly has been presented in^15^ wherein 3D surface scans of head have been acquired before and after treatment. The proposed statistical model has been used to monitor the progress of helmet therapy in infants. The approach of statistical shape modeling has been also used in craniosynostosis as the accurate results have been achieved. As an example, the given work in^16^ presented a pipeline in order to detect the craniosynostosis type based on statistical shape modeling and machine learning. One of the approaches based on head image analysis has been proposed in^17^ wherein photographic techniques versus flexicurve band measurement have been examined on infant heads followed by a comparison analysis. This was a substitution for physical measurement known as HeadsUp technique^1^. A bundle of valuable works has been presented in the framework of the project Shape-based Retrieval of 3-D Craniofacial Data mainly based on computer vision approaches (e.g.,^18^). The work presented in^19^ utilizes different regression models including regularized logistic regression, fused lasso and clustering lasso for the sake of skull retrieval in infants with craniofacial anomaly. The methods have been evaluated for classification of normal retrieved skulls from abnormal ones. Finally, the methods have been used for quantifying the skull shape before and after surgery based on the respective CT images. In another work given in^20^, the RGB images together with the texture parts are used in order to extract the face in infants with craniofacial anomaly. Standard machine learning methods like SVM and computer vision techniques like classic 2D face detection are utilized for this sake. Minimum accuracy of 91% has been reported for each building block of the whole extraction pipeline. The work presented in^5^ uses standard pipeline of 3D craniofacial image analysis by utilizing 3D and 2D image data followed by feature selection to feed the classifier. The image processing module includes 3D mesh and 2D exterior contour extraction to feed. The selected features are low-level features individually and in a combined form which feed the classification module consisting of an SVM-based classifier and logistic regression. The classification task classifies three types of synostosis skulls. The accuracy varies depending on the feature types wherein the highest accuracy of 99.2% has been reported. In another work presented in^21^, the craniosynostosis type classification based on the curvature distribution is given. In the latter work, the skull curvature is estimated using skull segmentation based on the extracted surface generated from 3D skull scans. The curvature is compared with the one of normal skull shapes to classify different types of skull shape variation. Using deep learning and pretrained network to elaborate the feature selection and extraction task has been a common practice since years ago. For example, in the recently new work presented in^3^, transfer learning technique is utilized to perform sagittal craniosynostosis classification. The proposed method uses pretrained network Google Inception V3 model given by Google under the TensorFlow design^22^. The network is fed by labeled image data from CT slices each of resolution $512 \times 512$ pixels. The achieved accuracy on the extracted dataset reported is 95%. The latter approach indicates the outperforming the deep learning methods over the traditional hand-crafted feature approaches which is expected upon the utilizing the deep structure normally. Besides, there are some works based on the developed smartphone Apps/tools aiming at cranial deformation analysis. For example in the work presented in^23^, a smartphone app is used to guide ordinary (non-clinical) users to capture several images from the infant’s head in order to register the image. At least 200 images are required by the model while the subject (patient infant) is not moving. The infant’s head is covered by a coded cap which is added by 131 markers which allows quick detection and identification. The recorded frames of the head covered by the cap are utilized to register a 3D model of the head. The final 3D model is extracted in the server side after receiving all of the required data. The achieved accuracy (deviation) is less than 1.5 mm in computing longitudinal and transversal and primeter measures making the model a suitable one for clinical purposes^23^. In another approach presented in^24^, deformational plagiocephaly and branchycephaly parameters are measured using devices like smartphones or tablets. The proposed approach utilizing advance imaging algorithms to detect different types of the latter deformational severity from top-view photos also can be used by non-expert people. The imaging techniques are used to extract the head counters to measure the parameters Cranial Index (CI) and Cranial Vault Asymmetry Index (CVAI). The accuracy in terms of correlation between the ground-truth and the extracted measurements are 0.94 and 0.96.

## Methods

### Problems and challenges

Lack of standard methods to quantify the severity of deformational plagiocephaly in children is an important obstacle in monitoring and treatment process. The existing measurement methods (e.g., using a caliper) are subjective and depending on the experience of the trained domain expert. The latter concerns together with advances in computer vision and deep learning as well as software development motivated us to develop a mobile application for parents and physicians to quantify the shape of a child’s head from a bird’s eye view photograph. In this regard, the length, the width, the left and right transcranial diagonals as well as the CVAI are measured. In order to properly measure the head shape and anomaly in metric scales, a reference object is required. This object is placed in the central point on the vertex of the child’s head. The main challenges include the following:

- Detect reference object and calculate scaling
- Detect and segment head
- Measure anthropometric parameters such as cranial length, width, CVAI from segmented head

To tackle these challenges, we have tried different methods based on computer vision and deep learning for semantic segmentation of the region of interest. The method based on the deep learning network architecture will be described in the next section in detail. A data set consisting of patients consulted for craniofacial deformities at the Department of Cranio-Maxillofacial Surgery at the University Hospital of Giessen has been created at first. The project has been approved by the ethics committee of Justus-Liebig-University Giessen (Az. 143/16) and written informed consent was obtained from the legal guardians in all cases. Also, all methods were performed in accordance with the relevant guidelines and regulations. All patients consulted for cranial deformity by a specialist for cranio-maxillofacial Surgery in Giessen have been offered to participate in this project. The collected dataset for this study consists of 115 patients, all with head asymmetries of different severity grade. Images were captured at first visits as well as on follow-ups for already treated patients. If at least a moderate craniofacial deformity was diagnosed during the consultation and a therapy was aspired, the child’s head would scan with a stationary 3D-Scanner (Vectra 5-Pod-System, Canfield sci.). This process has been performed by taking five simultaneous photographs from different positions. To eliminate artifacts from the scan, e.g. caused by a child’s hair, the child is equipped with a wearable tight cap. After the 3D scan, the child is placed on her/his parent’s lap to be stabilized when capturing the photo. In the developed app, a profile is created for the respective child and filled with both personal data such as age and gender as well as information of disease history, the diagnose date (deformity detection) by the parents and the physician and possible initial therapy measures (if applicable). In the next step, the coin is placed on the vertex of the head covered by the cap. We have chosen a 50-Eurocent coin as the reference object for our dataset, due to its standardized size and extended availability in Euro zone. The images are captured from a bird’s eye view (top angle) with the app. In addition to the images we have recorded the data elements like length, width, circumference and the diagonals for each patient. The manually measured parameters were taken as the gold standard for the validation of our developed mobile application. The patients were between 90 and 824 days old. The lower-bound and upper-bound values of the recorded parameters of patients are presented in Table 1.

**Table 1:** Range of the recorded parameters of the studied patients collected by anthropometric caliper measurement.

Parameters	Lower-bound (cm)	Upper-bound (cm)
Circumference	39.2	51
Length	12	17
width	11.3	14.5
Diagonal A $40^{\circ }$	12.1	15.9
Diagonal B $40^{\circ }$	11.7	16.9
CVAI	0.1	2.6

It is worth to mention that we have initially approached the aforementioned problems via a classical computer vision and image processing techniques as well. The detection of the reference object has been implemented using the Hough Circle Transformation (HCT). After image pre-processing, the local thresholding with empirically estimated thresholds and erosion operation have been performed to delete outlier pixels in the region of interest of the coin. Using HCT, the coin area has been segmented. In order to measure the head area, the ratio between the head and coin has to be detected as a feature. In this regard, the center and the radius of the detected HCT-circle are localized and measured. The accuracy of the model has been evaluated using 275 images as well wherein the overall accuracy of 83.63% in two sequential phases of coin and head detection has been achieved. The individual phase accuracy is 95.64% and 87.45% respectively. As the main method which is introduced in the next section outperforms the latter one significantly, we have skipped the detail description.

### Algorithm description

In order to speed up learning and to improve the accuracy of our segmentation model, we have taken the advantage of transfer learning. The model has been initialized with pretrained weights from the 2012 ILSVRC ImageNet dataset^25^. The model has been trained using Adam optimizer using dice loss with adjusted class-weights, emphasizing the coin and head classes, combined with a categorical focal loss^26^. As evaluation metrics Intersection over Union, also known as Jaccard index, and F1-score have been selected. The general description of training process is presented in Algorithm 1 below.

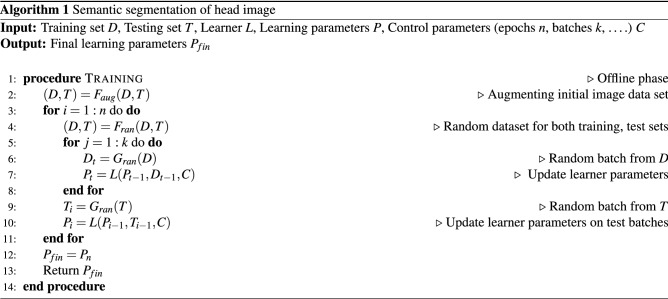



## Results

### Architecture and data augmentation

**Figure 1:**
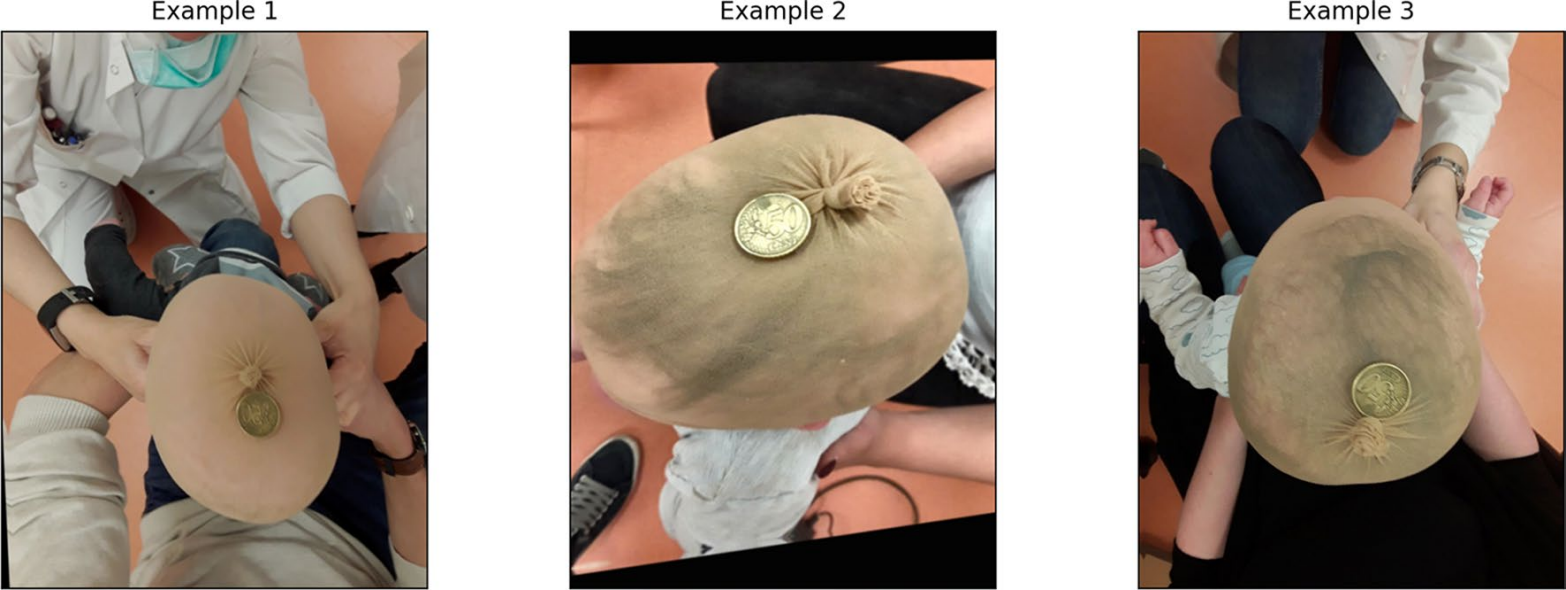
Example of different image augmentation on a randomly drawn image from the training set.

In our proposed approach, transfer learning through pretrained network is used to semantically segment the regions covered by the head and coin (used for measure reference) respectively. The main advantage of current method over the feature-based approaches is that the feature extraction and selection is done by the network which results in an elaborate feature combination set. These can be applied to a specific use case based on the customized dataset. The underlying network is organized as a U-Net^27^, which is an encoder-decoder architecture for segmentation models. The established model contains four encoder layers, which performs downsampling and encoding the images consecutively. The encoder inputs are concatenated with the respective decoder outputs after upsampling to maintain spatial information about the detected features. Encoder and decoder layers are residual convolutional blocks of ResNet-18^27^ type. The available dataset has been split into train and validation sets with a ratio of 2:1. Afterwards, all available images have been downscaled by a factor of 3.4 and zero-padded to a final input size of $736 \times 960$ pixels to be divisible by 32, as the requirement of the network architecture. The data augmentation then has been applied to the training data to end up with a final training set size of 498 images. Applied augmentation techniques include rotation, horizontal flip, additive Gaussian noise, random manipulation of different image features, e.g. contrast or brightness and more. A full list of applied augmentation operations with the corresponding occurrence likelihood is listed in Table 2. Also, an example of applying augmentation operations on the original image is shown in Fig. 1.

**Table 2:** A summary of the augmentation operations.

Augmentation operation	Parameters	Likelihood value
Horizontal flip	–	0.5
Rotation	[$-\,90^{\circ },\, +90^{\circ }$]	1.0
Gaussian noise	Mean: 0 Std: 0.01, 0.05	0.2
Contrast adjustment	4.0, $8 \times 8$	0.9
Adaptive histogram equalization	–	
Random brightness	[− 0.2; + 0.2] [80; 20]	
Random Gamma	[80; 20]	
Random Contrast Hue Saturation Value	[− 0.2;+0.2] [H: (− 20, +20); S: (− 30, +30); V: (− 20, +20)]	0.9

### Accuracy analysis and evaluation measures

**Figure 2:**
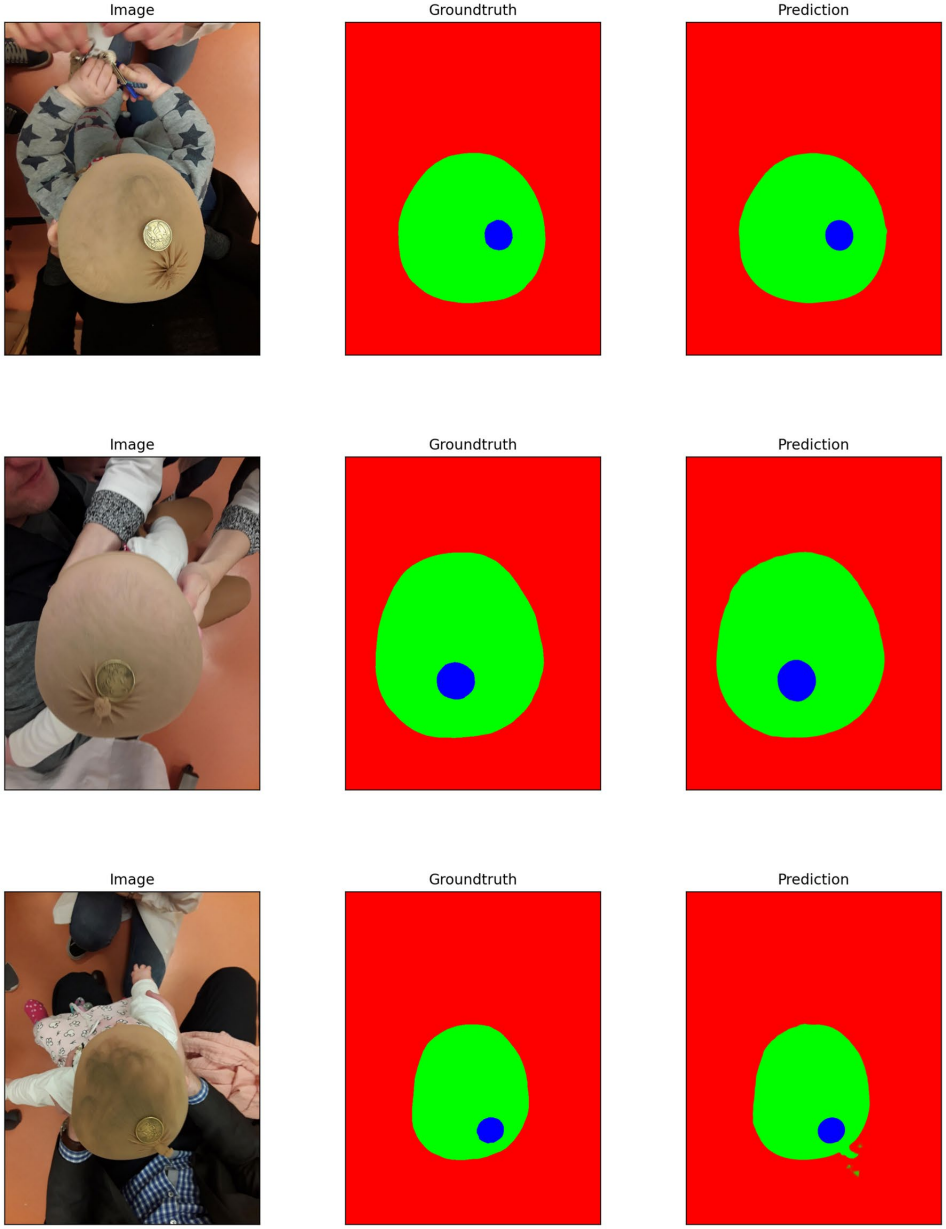
Examples of images with applied segmentation technique. The first image from top contains the coin almost in the middle and the segmentation result is done perfectly. In the second image the coin is not aligned with the center of the head although the segmentation is performed impeccably. The last image (image in bottom contains the coin connected with the tie of the covering head so, some outlier are detected falsely.

To evaluate the accuracy performance of the method, all classification parameters including elements of the confusion matrix and extracted parameters, and corresponding Receiver Operating Characteristic (ROC) curve are presented. As the second approach outperforms the first one, we are focusing on the accuracy analysis of the second approach wherein more elaboration has been made as well. An example of original images (captured by the user) as well as the final segmentation results have been presented in Fig. 2. In this figure, three examples of the original images captured by the smartphone camera are shown in the left side. The manually detected Region Of Interest (ROI) including the head and reference coin objects of the corresponding images are shown in the middle column. Finally at right, the detection results of the prediction model are displayed. According to the figure, the prediction results in the first two examples are almost perfectly matched with the manual ones even when the coin is not placed exactly in the center. In the third image example, the reference coin is bottom-right aligned. So, the detection result in the latter case suffers from connecting the head and background of the image.

**Figure 3:**
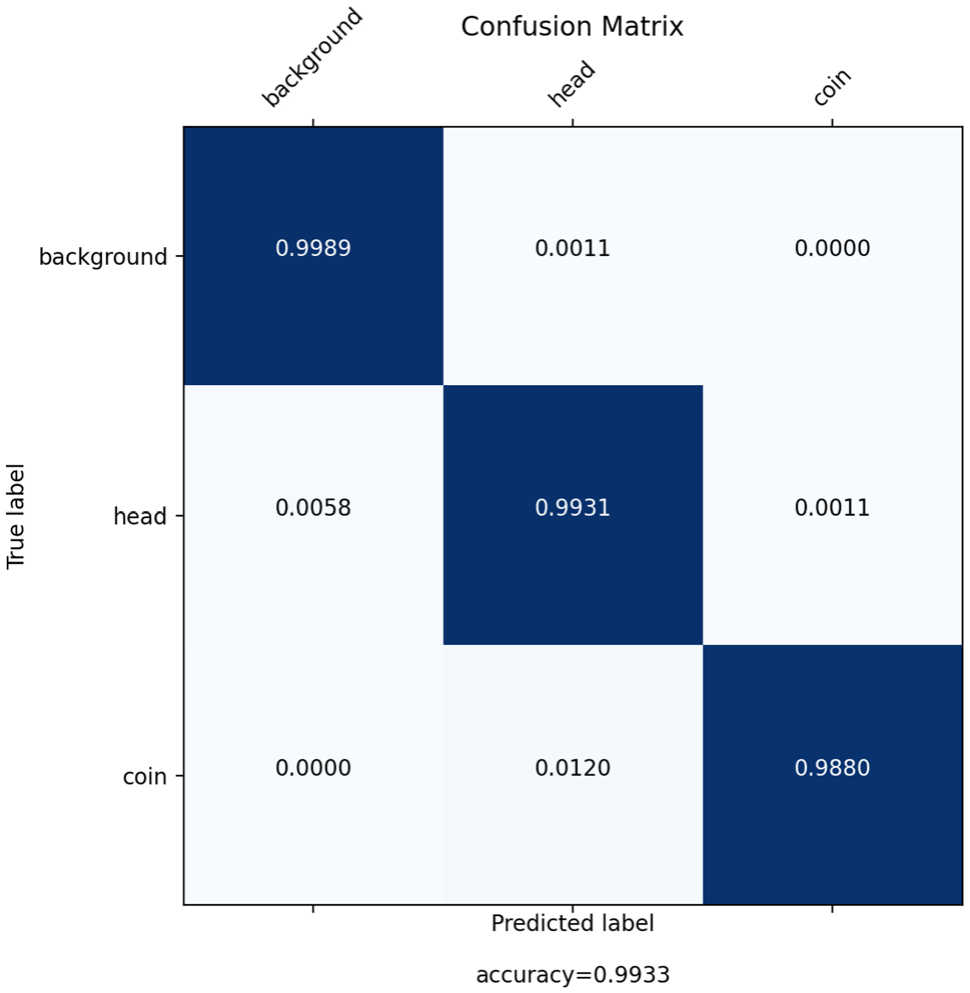
Confusion matrix corresponding to the main semantic segmentation model.

To elaborate and improve the accuracy results, the model has been furnished through data augmentation. To assess the model accuracy, the finalized training set of 498 augmented and re-scaled images are utilized. The details of the applied operations on the original images to get the augmented image set has been described in the last section. The train set contains 498 images. The final model has been tested via 28 images. The summarized results of the model accuracy are presented in the confusion matrix in Fig. 3. The entries of a confusion matrix include the corresponding True Positive, True Negative, False Positive and False Negative occurrences.The true detection entries are located in diagonal cells while the false detection entries are located in off-diagonal cells of the matrix. So, the higher the diagonal and lower the off-diagonal entries, the higher will be the classification performance.

Also, ROC curve and Precision–Recall curve have been extracted. The Receiver Operating Characteristic curve is a plot that displays the trade-off between the True Positive Rate (TPR) or sensitivity and False Positive Rate (FPR) or 1-specificity. The components are computed as follows wherein TP, P, FP and, N stand for True Positive, Positive, False Positive and, Negative respectively.

$$\begin{aligned} TPR=\frac{TP}{P} \quad FPR=\frac{FP}{N} \end{aligned}$$

The Precision–Recall curve displays the trad-off between the Precision and Recall (True Positive Rate). The definition of Precision is as follows wherein FP stands for the False Positive:

1 $$\begin{aligned} Precision=\frac{TP}{TP+FP} \end{aligned}$$

**Figure 4:**
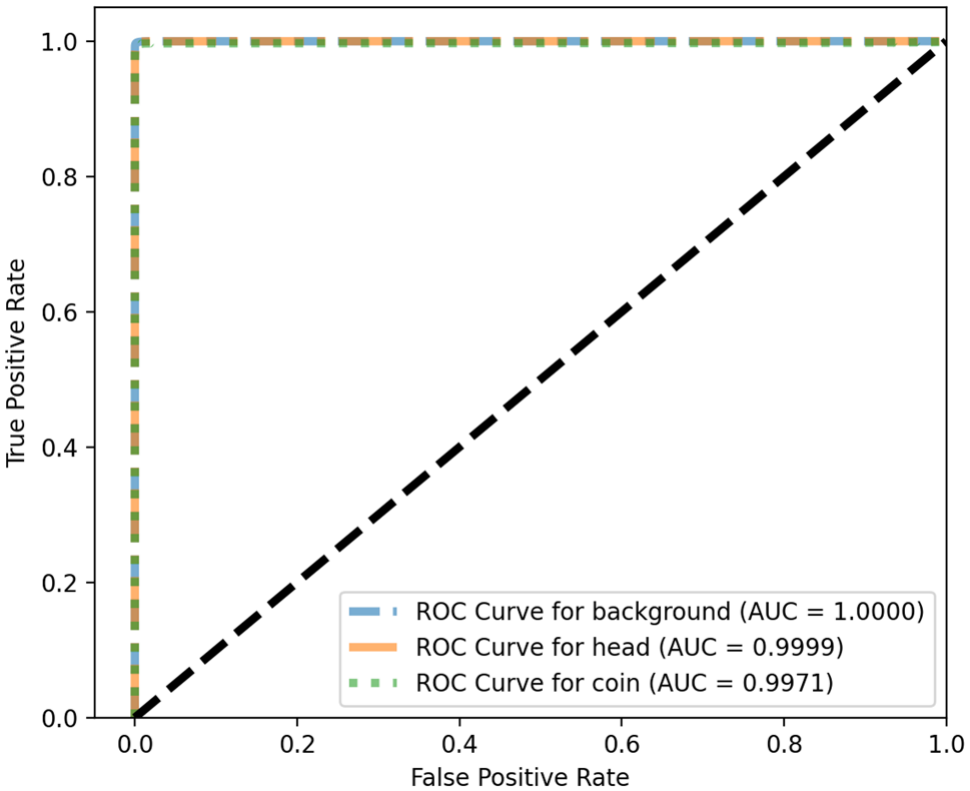
ROC curves corresponding to the 3-label classification problem.

**Figure 5:**
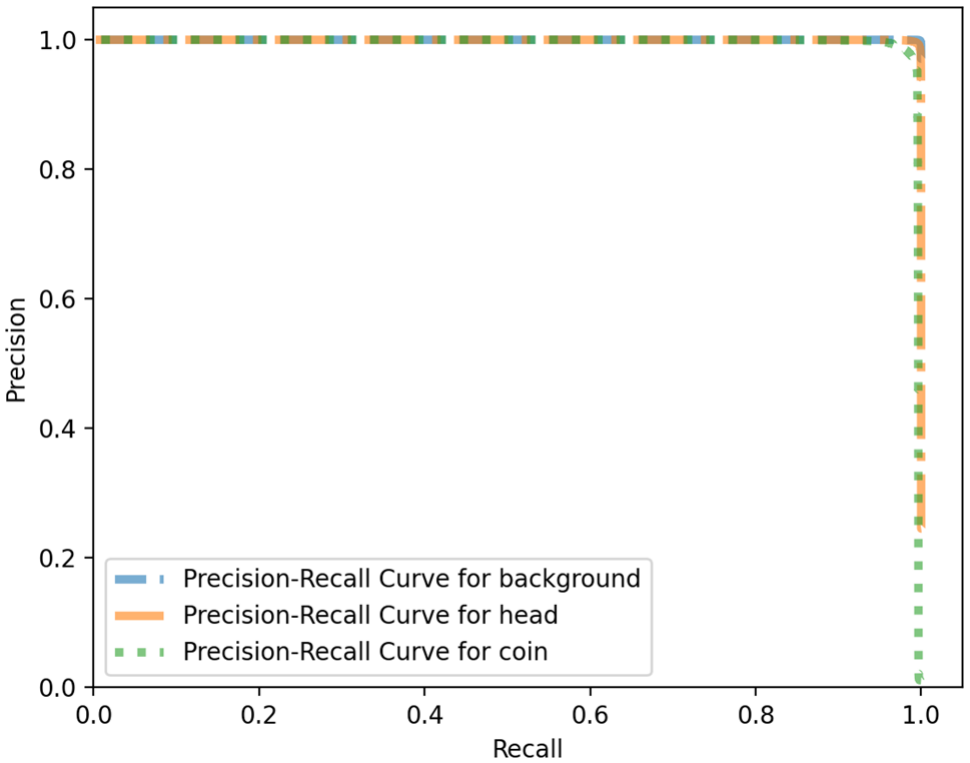
Precision recall curves corresponding to the 3-label classification problem.

**Figure 6:**
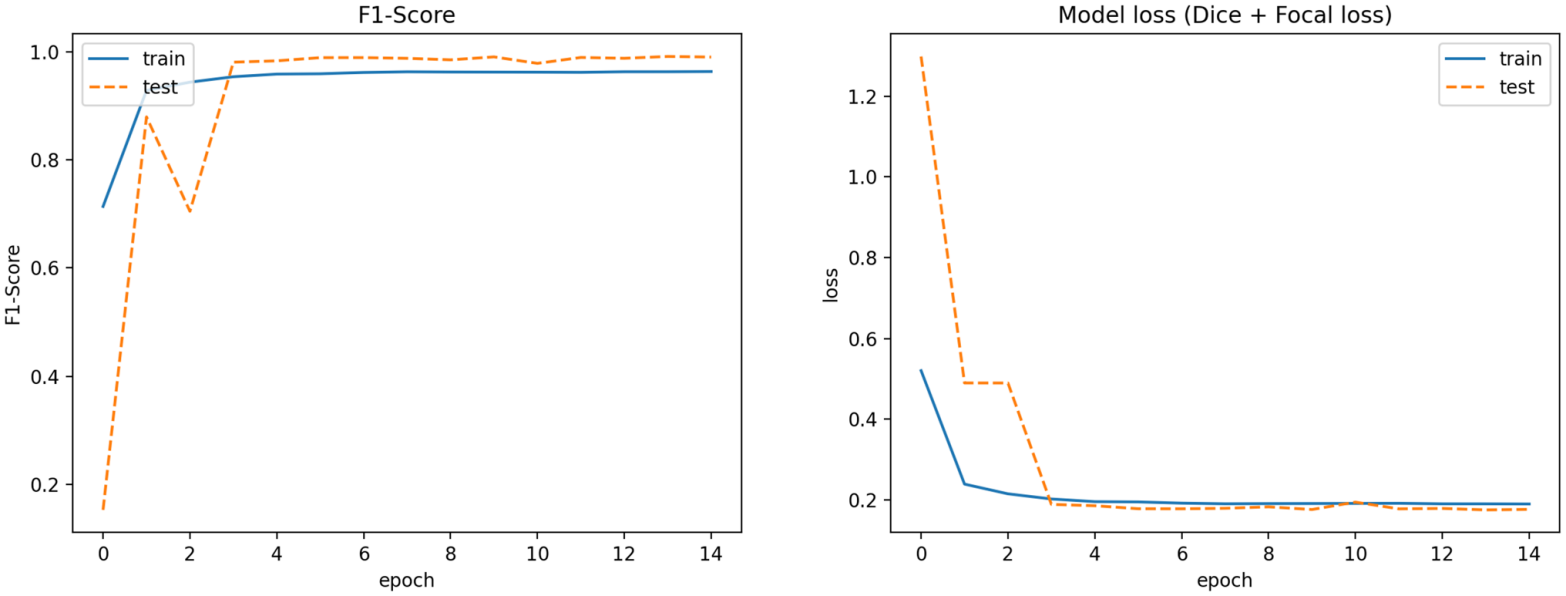
The trend of network convergence in terms of F1-score and corresponding loss function.

The curves and the corresponding Area Under the Curve (AUC) are as follow in Figs. 4 and 5 respectively. A high area under the curve in a ROC curve indicates high discriminating capability of the model in distinguishing between the binary classes. A high area under the curve in a Precision–Recall curve represents both high measures. The network converges very fast and high F1-score as well as low loss value are achieved. The convergence trend is shown in Fig. 6.

## Discussion

### Implementation and development

**Figure 7:**
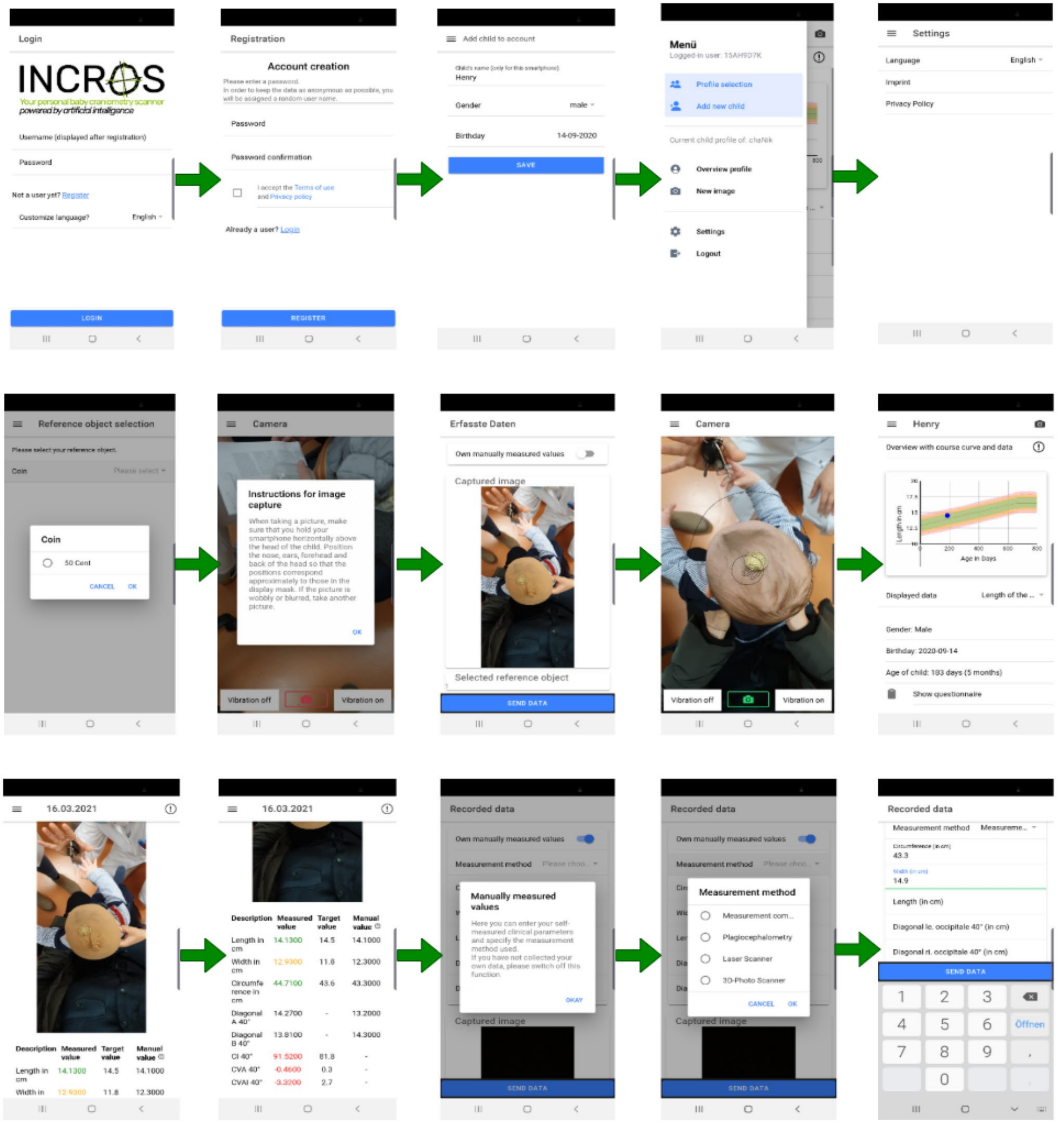
Some screenshots of the developed app from users side (from top-left to bottom-right): at first, user opens the app. After creating an account and filling out the questionnaire regarding to the corresponding medical history of the kind, the user will be asked to choose the reference coin and capture the photo of the baby’s head. The head as well as coin will be then detected and the corresponding measurement parameters will be calculated and stored. The option for manual calculating and storing the measurement parameters is also available just in case. Finally the measurements will be submitted to the requesting party.

The proposed approach has been implemented and the classification parameters including the corresponding analysis have been quantified. The semantic segmentation model was implemented and trained using Python (Version 3.7.5), Keras (Version 2.2.4) and Tensorflow (Version 1.14.0). The leveraged U-Net architecture and the ResNet-18 building blocks are implemented in Python segmentation-models package (Version 1.0.1). All augmentation techniques were applied using albumentations^28^. The finalized model was transformed to tensorflow-js using the official tensorflow-js converter from the python package. The model will be hosted on an webserver in order to be accessible by the Ionic 5 hybrid application for the sake of segmentation (the codes and data are available upon request). Concerning the widespread usability of smartphones, their ability to generate high quality images and to establish a commonly-used and cost-efficient home-based platform, a smartphone app has been developed. Using this app, the users can easily capture the photo of the baby’s head through the internal camera of smartphone. The app will detect the head and report the required measures in real-time. The data will be stored in patient’s profile. The general pipeline of the app from users side including the features is shown in Fig. 7.

### Statistical analysis

**Figure 8:**
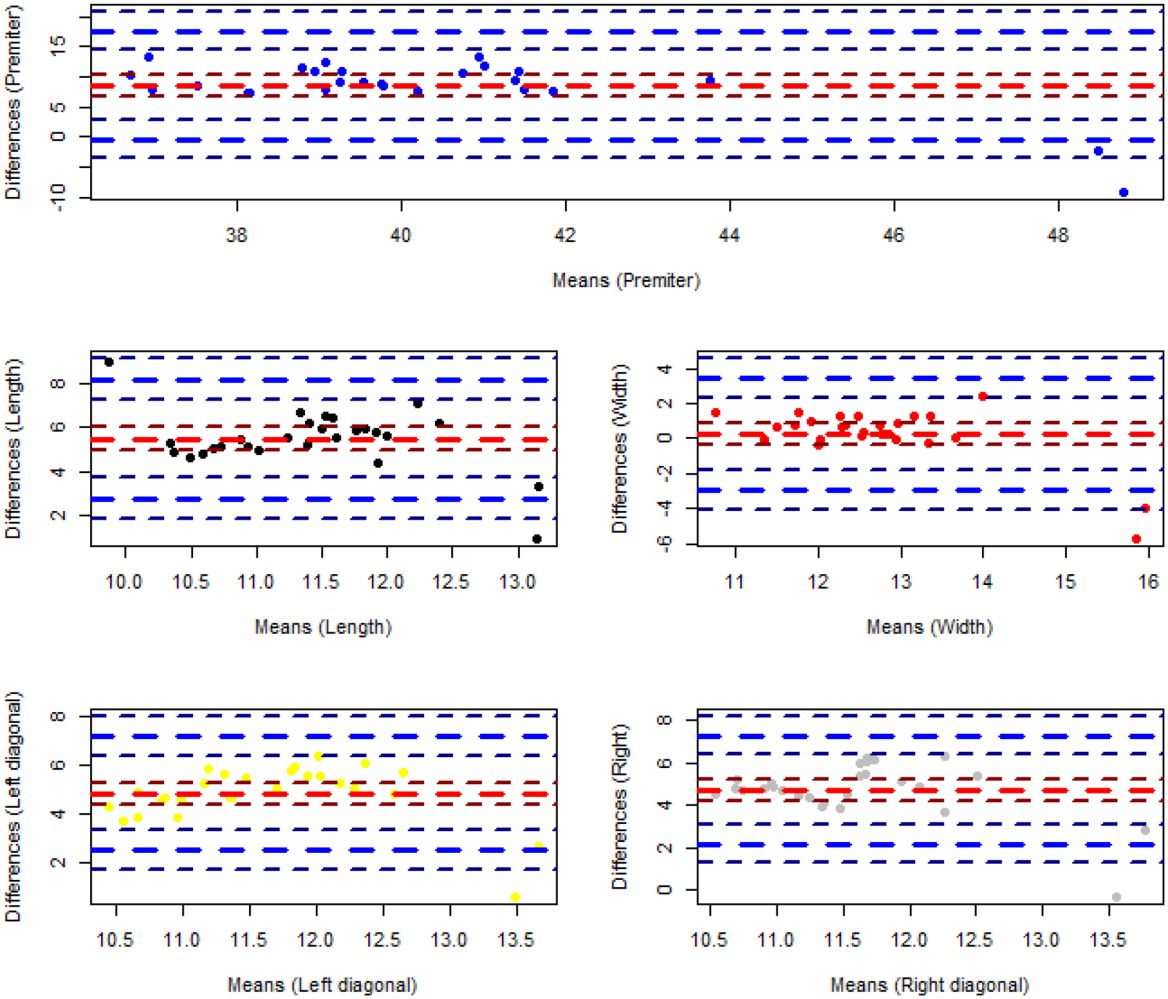
Representation of errors of clinical parameters calculation (the red dash line presents the mean and the blue dash lines present the variance of the differences).

The deformity parameters of all detected segmented regions in the test images are calculated based on the segmented areas. Five parameters as height, width, perimeter and, two diameters are calculated which can be used as the indicators for the physicians. The error analysis has been performed on the acquired measurements of the test data set. The observed measurements on the test data indicate the systematic type of the errors introduced into all five measurement parameters as depicted in Fig. 8. So a non-linear regression technique has been decided for the final measurement correction. Five non-linear models have been fitted to the data as $Y = \beta _{0}+ \beta _{1}X+\beta _{2}X^{2}$ and the results are shown in Table 3.

**Table 3:** Regression models parameters.

Parameters	$\beta _{0}$	$\beta _{1}$	$\beta _{2}$
Height	11.3263	0.5256	− 0.0227
Width	− 8.3606	2.8362	− 0.0896
Perimeter	− 10.7224	2.6182	− 0.0295
Left diagonal	− 24.5070	7.2812	− 0.3314
Right diagonal	− 4.2913	3.4149	− 0.1534

**Figure 9:**
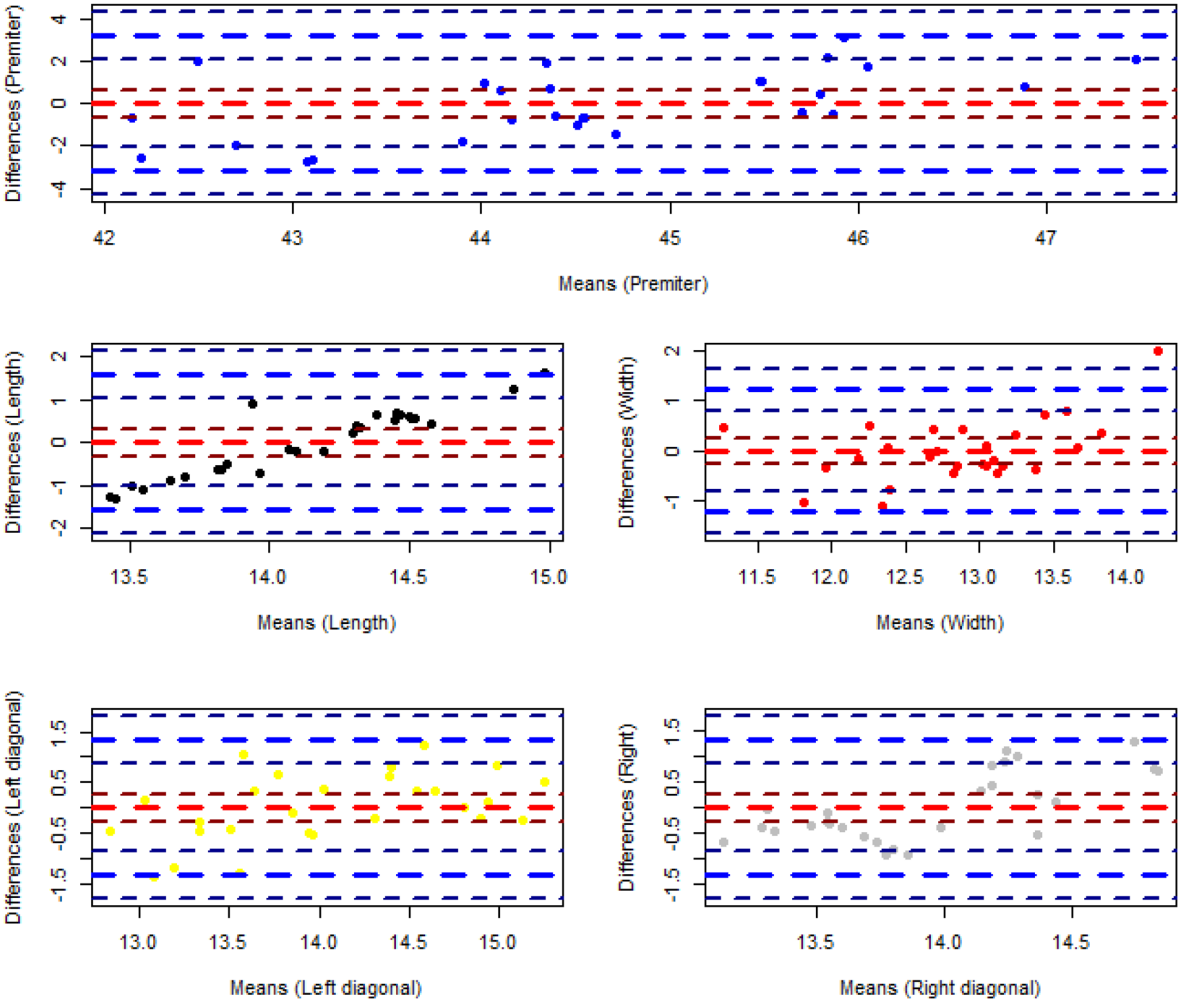
Distribution of the regression errors of the fitted values corresponding to five measurement parameters of the test data set (the red dash line presents the mean and the blue dash lines present the variance of the differences).

The distribution of the regression error (residual) between the manually measured values and measured values on the segmented areas by the model for all parameters is shown in Fig. 9 as well. As observed in the graph, the errors follow the normal distribution as it has been confirmed by Shapiro normality test as well according to the extracted P-values (all greater than 0.05). So, the confidence interval of 95% is set for the regression model. Also, the numerical values of Mean of Absolute Error (MAE) as well as Mean Squared Error (MSE) have been calculated as follow presented in Table 4. Both measures are utilized as indication to evaluate the closeness of the regression estimation of the clinical parameters to the corresponding real values. If all errors are treated equally then MAE measure is focused while MSE penalizes the higher regression error. Both error metrics have non-negative range and the lower error values imply higher accuracy of the regression model. According to Table 4 all of the quantified values are less than 0.74 cm except for Perimeter in which the Perimeter values are bigger than the other clinical parameters trivially. The relative minor regression error values make the extracted model an acceptable model to be used in practice. However, the model will be elaborated more while more clinical experiments and validation studies are conducted.

**Table 4:** Mean of absolute errors (MAE) of the measurement parameters.

Parameters	MAE (cm)	MSE (cm)
Height	0.6611	0.7357
Width	0.4544	0.6119
Perimeter	1.2942	1.4903
Left diagonal	0.5491	0.6740
Right diagonal	0.5434	0.6210

### Comparative analysis

To have a reasonable comparison between the presented work and the similar works in the literature the comparative analysis focuses on the works whose main method is based on 2D images captured by smartphone cameras. The available tools based on the smartphones have been mentioned and briefly described before. However, they are mainly using 3D imaging techniques. The recently presented tool called “Scully Care” app (https://en.skullycare.com/) is the most similar app to our app (INCROS) wherein the 2D images are captured through smartphone camera. The presented app calculates deformational parameters CI and CVAI from the extracted image to identify the abnormal severity. However, our presented app calculates CI, CVAI together with other deformational diagonal parameters. The validation study of the Scully Care App has started since 2019 while the validation study of our presented work through app has begun since March 2021. The measurements of our app are extracted and available in 11 seconds for the users and it is free of charge. The Scully Care app is commercialized and the results are available depending on the subscription type in a longer time. Our app supports two languages as English and German while Scully Care app supports 18 languages. Finally, from usability perspective, our work requires just a brown stocking cap and a reference coin for the infant. However, the aforementioned app requires a white cap, a blue mat, and a set of foam ear spreader before image acquisition.

## Conclusion

In this paper, a classification model based on a deep learning network architecture through transfer learning has been presented. The aim of our model is to perform semantic segmentation to detect and monitor abnormal skull shape in children with deformational plagiocephaly. We have implemented the method using Python and relevant libraries. The achieved accuracy of the model on the 3-class classification problem is 99.01% while the specificity and sensitivity are quantified as 99.46% and 98.94%, respectively. The model has been implemented and deployed and a mobile app has been developed. The developed app enables non-clinical users including parents to monitor the disease, prevent progression of deformity and control treatment progress at home and in an outpatient environment. Reports to clinical parties can be assured in a timely manner by the application. Also, the user achieves the measurement results latest in eleven seconds after image capturing by the app. The proposed application however suffers from some limitations. To capture the photo through the app, the children must wear a tight caps to cover their hair. Otherwise, the detection will be falsified by connecting the background patterns to the Region Of Interest (ROI) due to similar patterns or colors leading to mis-classification. Also, the model only measures the values basis on the two-dimensional images and thus only looks at the top half of the head from bird’s angle view. During consultations, the clinical parameters are measured slightly above the ears. These measurement points are not visible on the images with no depth, which can cause deviations. Augmenting the model with more inter-operable user interface, as well as an extensive monitoring and validation study increasing trustful usability of the tool are being planned as future work.

## Data Availability

The utilized clinical data as well as implementation codes are available upon request.
